# Are Flying-Foxes Coming to Town? Urbanisation of the Spectacled Flying-Fox (*Pteropus conspicillatus*) in Australia

**DOI:** 10.1371/journal.pone.0109810

**Published:** 2014-10-08

**Authors:** Jessica Tait, Humberto L. Perotto-Baldivieso, Adam McKeown, David A. Westcott

**Affiliations:** 1 School of Energy, Environment and Agrifood, Cranfield University, Cranfield, Bedfordshire, United Kingdom; 2 CSIRO Sustainable Land and Water, Smithfield, QLD, Australia; 3 CSIRO Sustainable Land and Water, Atherton, QLD, Australia; University of Tasmania, Australia

## Abstract

Urbanisation of wildlife populations is a process with significant conservation and management implications. While urban areas can provide habitat for wildlife, some urbanised species eventually come into conflict with humans. Understanding the process and drivers of wildlife urbanisation is fundamental to developing effective management responses to this phenomenon. In Australia, flying-foxes (Pteropodidae) are a common feature of urban environments, sometimes roosting in groups of tens of thousands of individuals. Flying-foxes appear to be becoming increasingly urbanised and are coming into increased contact and conflict with humans. Flying-fox management is now a highly contentious issue. In this study we used monitoring data collected over a 15 year period (1998–2012) to examine the spatial and temporal patterns of association of spectacled flying-fox (*Pteropus conspicillatus*) roost sites (camps) with urban areas. We asked whether spectacled flying-foxes are becoming more urbanised and test the hypothesis that such changes are associated with anthropogenic changes to landscape structure. Our results indicate that spectacled flying-foxes were more likely to roost near humans than might be expected by chance, that over the period of the study the proportion of the flying-foxes in urban-associated camps increased, as did the number of urban camps. Increased urbanisation of spectacled flying-foxes was not related to changes in landscape structure or to the encroachment of urban areas on camps. Overall, camps tended to be found in areas that were more fragmented, closer to human habitation and with more urban land cover than the surrounding landscape. This suggests that urbanisation is a behavioural response rather than driven by habitat loss.

## Introduction

By 2030 five billion humans are expected to live in urban areas while the global urban footprint is predicted to expand by 163% from c. 727,000 km^2^ to c. 1,527,000 km^2^ [1]. While already significant ecosystems in their own right, such urban expansion will place increasing pressure on other land uses, threatening native species and ecosystems, and becoming potent sources of invasive species and pathogens [2]. For example, much urban expansion is currently occurring in sensitive areas for biodiversity, e.g. in coastal lowlands or close to protected areas [1]. This means that consideration of urban systems is increasingly important in conservation planning and management. Urbanisation generally has a negative net effect on biodiversity, with many species becoming rare or locally extinct, in particular specialists, slow reproducers, and disturbance-sensitive species [2], [3]. However, some species, often generalists, readily adapt to the urban landscape, some even reaching higher abundances in cities than in natural vegetation [4], [5]. Although the presence of wildlife in urban areas can enhance human quality of life, some urban animal populations can prove problematic due to their impacts on amenity, damage or their role as vectors of disease [6], [7]. Understanding how particular species respond to urbanisation and identifying the processes leading to these responses is fundamental if we are to successfully manage the interaction between urbanisation, biodiversity and human welfare.

Urbanisation influences species distribution, abundance and movement. The urban mosaic can prove an attractive habitat for a wide range of taxa due to abundant food and shelter [2], [3], [8], [9]. Urban areas can provide a refuge from hunting or predation pressure [10], from environmental disturbances such as drought or fire, as well as a more stable resource supply whilst natural vegetation is recovering [11]. Wildlife adapt to urban areas in a variety of ways, e.g. by adjusting their foraging, anti-predator behaviour, breeding behaviour and taking advantage of the climate associated with urban areas [12], [13], [14], [15]. With increased urbanisation of wildlife populations comes increased contact with humans and attendant increases in opportunity for conflict, including amenity impacts such as noise, smell and vegetation damage [16], hazards such as vehicle collision or attacks on pets [17], [18], and risk of disease transmission [6], [7]. These ‘human-wildlife conflicts’ can lead to intense disagreement over the management of habitat and wildlife in and around urban areas [19].

Flying-foxes are large (up to c. 1 kg), colonially roosting bats which readily adapt to urban ecosystems. Roost sites (hereafter called camps) are found in and around many Australian towns and cities [20], [21], [22], [23]. Hypotheses for why flying-foxes might use urban areas include loss of native habitat and urban expansion [20], [24], changes in resource distribution due to plantings [25], [26], [27] and urban effects on local climate [21]. Evidence from other species suggests that urban areas may also provide refuge from predation [27], disturbance events such as droughts [28], bushfires [29] and post-cyclone effects [22], [30], [31]. It may also be possible that urban areas may be attractive because they offer a movement advantage, e.g. increase the ease of manoeuvring in flight due to the open nature of the habitat or ease of navigation due to landmarks and lighting, e.g. [32].

Roosting by flying-foxes in urban and peri-urban areas can result in contact and conflict with humans. The greatest concern is impact on amenity. It is not uncommon for flying-fox camps to contain 50,000 individuals [22], [27], but even much smaller camps can be potent point sources of noise, odour and faeces, particularly when they occur within metres of residences. While smaller camps are often tolerated, larger camps in particular become a focus of community disquiet.

Alongside impact on amenity are concerns about the risks of disease transmission [33]. Bats host a high diversity of viruses and some of these are of agricultural and human health significance [33], [34]. It would appear that in cases such as the paramyxoviruses (which include diseases such as measles, distemper, mumps, parainfluenza, Newcastle disease), these diseases have been present in bats for very long periods and have switched hosts to other mammals, including humans [34]. In Australia, there are two diseases of particular concern that are known to be carried by flying-foxes: Hendra virus and Australian bat lyssavirus. It has been suggested that the dynamics of diseases such as Hendra in flying-fox populations and the pattern of spillover events from flying-foxes to horses, may be influenced by urbanisation through the effects it has on habitat loss and through that, on resource distribution, connectivity between groups of flying-foxes, and increased interactions between flying-foxes and horses [35].

The negative impacts of flying-foxes, and the ever more strident calls for their ‘control’ that these impacts create, are at odds with the significance of their ecological role and conservation status. Flying-foxes are significant pollinators and seed dispersers in most vegetation types in their range [24], [36]. Furthermore, two species, the spectacled flying-fox (*Pteropus conspicillatus*) and the grey-headed flying-fox (*P. poliocephalus*), are listed as vulnerable under Australia’s Environment Protection and Biodiversity Conservation Act (1999). The resultant divergent and strongly held opinions on flying-foxes creates persistent tension between those who wish to see the animals conserved and those demanding they be controlled. Flying-fox management is a contentious and politicised issue in Australia.

To date, despite urbanised flying-foxes being a major management issue in northern and eastern Australia, there are few studies of urbanisation of flying-foxes. Identifying whether urbanisation of flying-fox populations is in fact occurring and the nature of its drivers is a fundamental step in developing effective management solutions. The aim of this study is to determine whether spectacled flying-foxes are urbanising and whether landscape features or change are associated with this. We use data from 15 years of monitoring of the spectacled flying-fox population to examine the spatial and temporal patterns of association of spectacled flying-fox camps with urban areas in the main part of their Australian range, the Wet Tropics of north eastern Australia. Specifically we (1) ask whether spectacled flying-foxes are becoming more urbanised, (2) we test the hypotheses that any shift to urban areas is associated with anthropogenic changes to landscape structure or to an increase in the size or number of urban camps, and (3) whether the landscape characteristics of camp sites differ from those of the surrounding landscape and how this has changed over the study period.

## Materials and Methods

### Ethics Statement

This research was conducted under Animal Ethics Approvals from the CSIRO Ecosystem Sciences Animal Ethics Committee and complied with the Australian Code of Practice for the Care and Use of Animals for Scientific Purposes (2004; 2013). The work required no interaction with or handling of flying-foxes. The research was conducted under Scientific Purposes Permit #WTK03462308 and a 173P Authorisation from the Queensland Parks and Wildlife Service. The research was conducted on 50 separate land tenures. Details of the location of each camp have been lodged as a part of the National Flying-Fox Monitoring Program (NFFMP; http://www.environment.gov.au/node/16393) and camp locations, tenure, access information and camp sizes can be obtained from either from the NFFMP or from the authors.

### Study Species

The spectacled flying fox is a phytophagous species, feeding primarily on floral resources and fruits in a wide range of vegetation communities, including closed forest, gallery forest, eucalypt open forest and woodland, coastal Melaleuca swamps, mangroves, vegetation in urban settings, and commercial fruit crops [37]. This highly mobile species forages at night and can disperse seeds and pollen over large distances [24]. By day the animals roost in camps, with an unknown proportion roosting solitarily or in small groups throughout the year [22]. In Australia, the spectacled flying-fox is found in the Wet Tropics of Queensland World Heritage Area between Townsville and Cooktown with small outlier populations north in the Iron and McIllwraith Ranges on Cape York [22], [24] and to the south at Finch Hatton, near Mackay [38].

### Study Site

**Figure 1:**
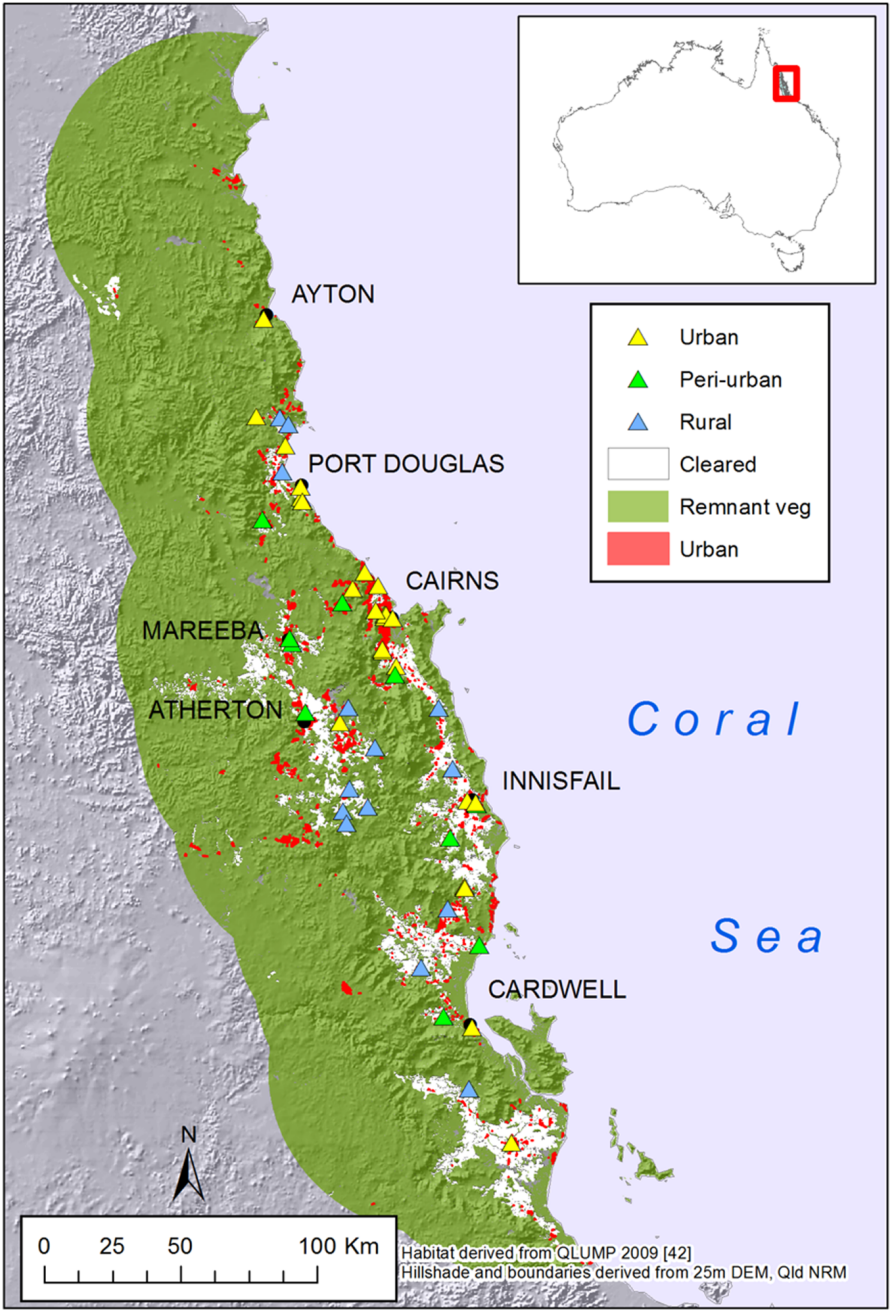
Location of the study area in the Wet Tropics Region of Northern Queensland, Australia. Spectacled flying-fox camps (triangles), towns (black dots) and urban areas (red shading) are also shown. Habitat mapping is derived from QLUMP 2009 [42] and the hillslopes and shading from the Qld. Dept. Natural Resources and Mines 25 m DEM (http://www.dnrm.qld.gov.au/mapping-data/data/topographic).

The study included all known current and past spectacled flying-fox camps in the Wet Tropics region of Queensland, an area of approximately 9,000 km^2^, on the north-east coast of Queensland (Figure 1). The region has a diverse, fragmented terrain of coastal plains and extensive uplands, typically 600–900 masl [39]. The vegetation is a complex mosaic of closed canopy rainforest and open eucalypt woodlands, with tropical savannas and grasslands on its drier margins and with clearing for agriculture on the wet fertile coastal floodplains, mid-montane tablelands and on the drier western slopes [39]. The region has a human population of approximately 252,000 [40]. Urbanisation is focused on several regional centres, Cairns, Mareeba, Atherton, Innisfail and Ingham, but there are also numerous small communities throughout the region.

### Camp data

Data on the location and sizes of all flying-fox camps in the region were obtained during regular monitoring programs begun in 1998 and continuing today. Over the course of the study the number of camps surveyed increased from 30 to 50 as new camp locations were identified. This increase resulted largely from the inclusion of historical camps. Once identified camps were not dropped from the surveys. These surveys were conducted under three programs. In 1998 and 1999 surveys were conducted in March and November while from 2000 to 2003 surveys were conducted in November only. These surveys were conducted by the Queensland Parks and Wildlife Service and involved positioning counters around the perimeter of camps to count the animals as they flew out of the camp at dusk, i.e. fly-out counts (see [41] for a more detailed description). Since May 2004 monthly, daytime, walk-through surveys of every camp in the study region have been conducted. In small camps (<1000 individuals), surveyors attempted to count all flying-foxes in a camp. In larger camps the density of individuals was assessed by counting the number of roosting individuals in randomly-selected roost trees, the average of these was then extrapolated to give a camp size estimate by counting the number of roost trees. Each regional survey was typically completed within three consecutive days to minimise the chance of inter-camp movements and any resultant recounting of individuals [22]. The use of these different survey methods in the early and later phases of the data collection has the potential to introduce biases into the data. To avoid such issues our analyses do not rely on direct comparisons of abundance estimates derived from different methods. This is achieved either by restricting analyses to data derived from a single method or by calculating proportions based on samples collected using a single method, i.e. within years, and only comparing the proportions across years.

### How urbanised is the spectacled flying-fox population in the Wet Tropics?

Count data from 1998–2012 was analysed to assess the proportion of the population associated with urban areas. Camps were assigned to three categories (urban, peri-urban and non-urban) depending on their distance from urban areas – defined as human habitation. Urban camps were defined as those surrounded by urban land use according to Queensland Government’s land use classification (primary attributes), QLUMP 2009 [42] and on-ground assessment. Peri-urban camps were defined as those adjacent to urban land cover. Non-urban camps were those more than 250 m from any urban land use - a distance at which there is usually little concern about the presence of flying-foxes. When referring to urban and peri-urban camps together we use the term urban-associated.

To examine patterns of urbanisation across years we used data from all surveys, i.e. from 1998–2012. To examine seasonal patterns of camp use we restricted the analyses to the monthly data obtained between 2004 and 2012. To detect any seasonal trends in urbanisation, the monthly percentage of the population in urban-associated camps, consistency of occupation (proportion of surveys in which a camp was occupied), mean camp size and total population surveyed were analysed using monthly survey data from 2004 onwards. The proportion of the population using identified camps is known to vary through the year with a lower proportion in camps mid-year and a higher proportion during the warmer months when mating, birthing and raising of young occurs. We use the term population to refer to that part of the population using camps at any point in time.

### Are spectacled flying-foxes being driven into urban areas by landscape change?

There were two QLUMP land use sampling periods during the time of the study, 1999 and 2009. These two sampling periods fell conveniently near the beginning and end of our study and so were used to document changes in land cover and use during the study period. QLUMP primary landuse attribute “intensive uses” was used as the first filter to determine urbanisation and the tertiary attribute was used to differentiate between non-urban uses, urban and rural residential uses. Percentage land cover assigned to particular land uses was extracted from QLUMP and overlaid with Regional Ecosystem data describing vegetation types [42] to produce the land cover categories: True urban, Rural Residential, Cleared, Rainforest, Sclerophyll and Other.

To determine whether areas surrounding camp sites had changed over time, landscape metrics which convey key information about landscape spatial structure were calculated in Fragstats 4.0 [43] for circular buffer zones with a diameter of 3.3 km around camps, this being half the average nearest neighbour distance between camps. These metrics were chosen to provide a description of the structure of the landscape in which camps occurred and were: forest mean patch area (MPA) a measure of the average size of forest patches in the buffer, forest patch density (PD) or the number of patches in the buffer, edge density (ED) or the length of patch edge as a function of area and a measure of the amount of interior patch habitat relative to edge habitat, and percentage urban cover (% urban) [44]. These measures for 1999 and 2009 were compared using Mann-Whitney U tests. In order to test whether camps were found in more fragmented areas than would be expected by chance, buffers around camps and n = 33 random points in the landscape were compared with Mann-Whitney U tests.

## Results

**Figure 2:**
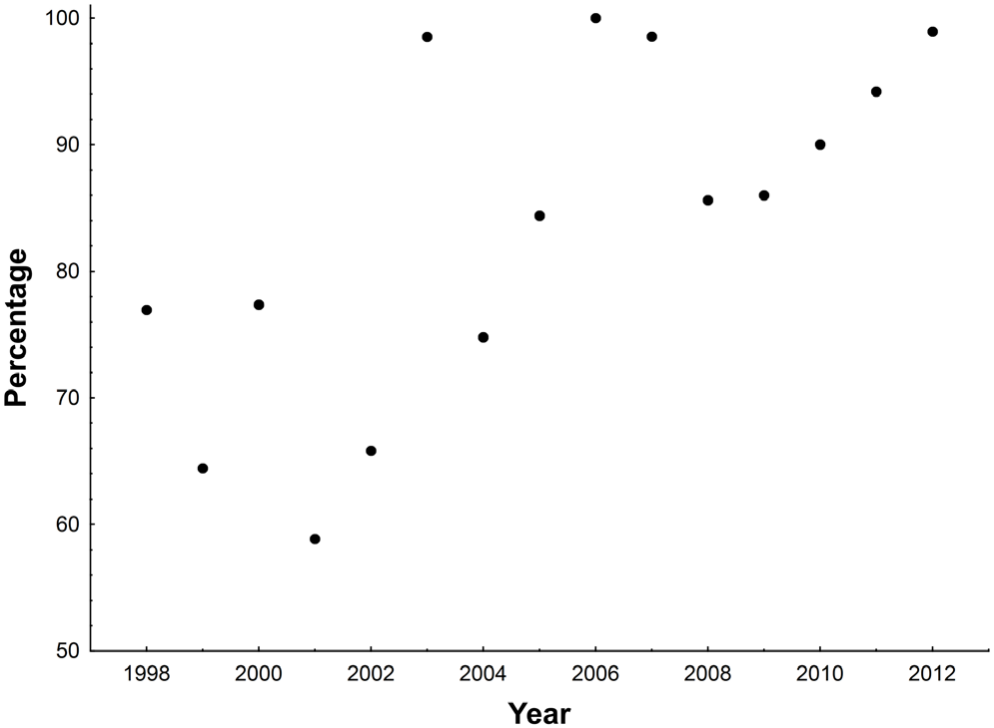
The percentage of the spectacled flying-fox population of the Wet Tropics found in urban associated camps during November surveys in each year of the study.

Surveys of spectacled flying-fox campsites across the Wet Tropics of Queensland from 1998–2012 indicate that the majority of the population were roosting in camps associated with urban areas (Figure 2). In each monthly survey between 5 and 16 camps were occupied (mean = 10, S.D.  = 2.3). While a total of 30 camps were surveyed in the initial years and this number increased to 50 in the final years of the monitoring there was no significant increase over time in the number of camps found to be occupied each year (r_p_ = 0.38, p = 0.16, n = 15) (Table S1 in File S1). There was an increase over time in the number of urban camps occupied each year (r_p_ = 0.64, p<0.01, n = 15) but no significant trend in the numbers of peri-urban or non-urban camps (p>0.05 for both). The net result was an increase in urban-associated camps (r_p_ = 0.5, p<0.03, n = 15). The mean percentage of the population found in urban and peri-urban camps across the 111 surveys was 82% (±26 S.D.), though this varied across all surveys from 59% in the early years to 99% in the later years.

**Figure 3:**
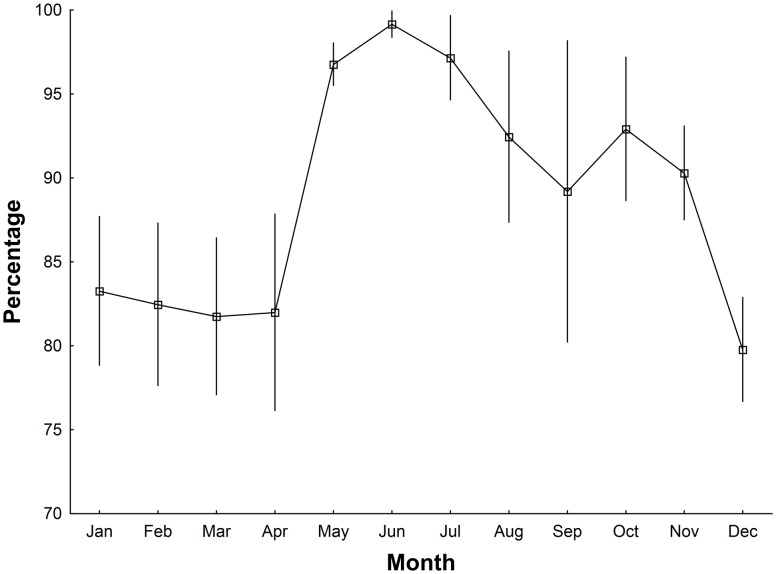
Changes through the year in the percentage (±S.E.) of the population occupying urban-associated camps. Monthly means calculated with survey data from 2004–2012.

**Figure 4:**
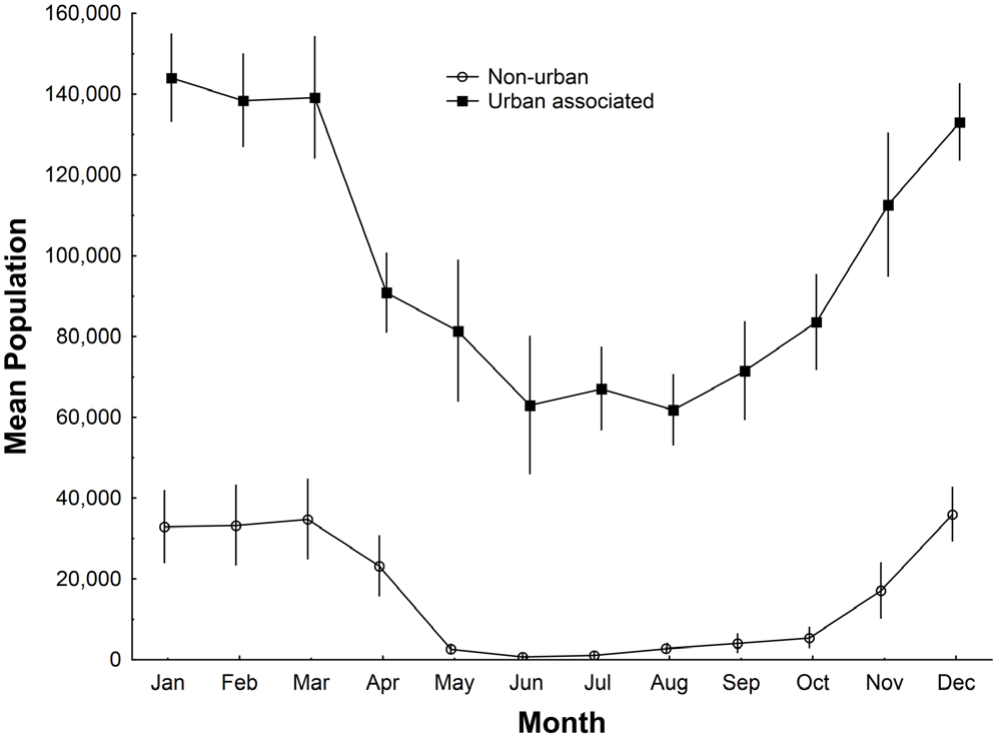
Changes through the year in the mean monthly population occupying urban associated and non-urban camps across the Wet Tropics calculated from 2004–2012 data. Monthly means calculated with survey data from 2004–2012.

**Figure 5:**
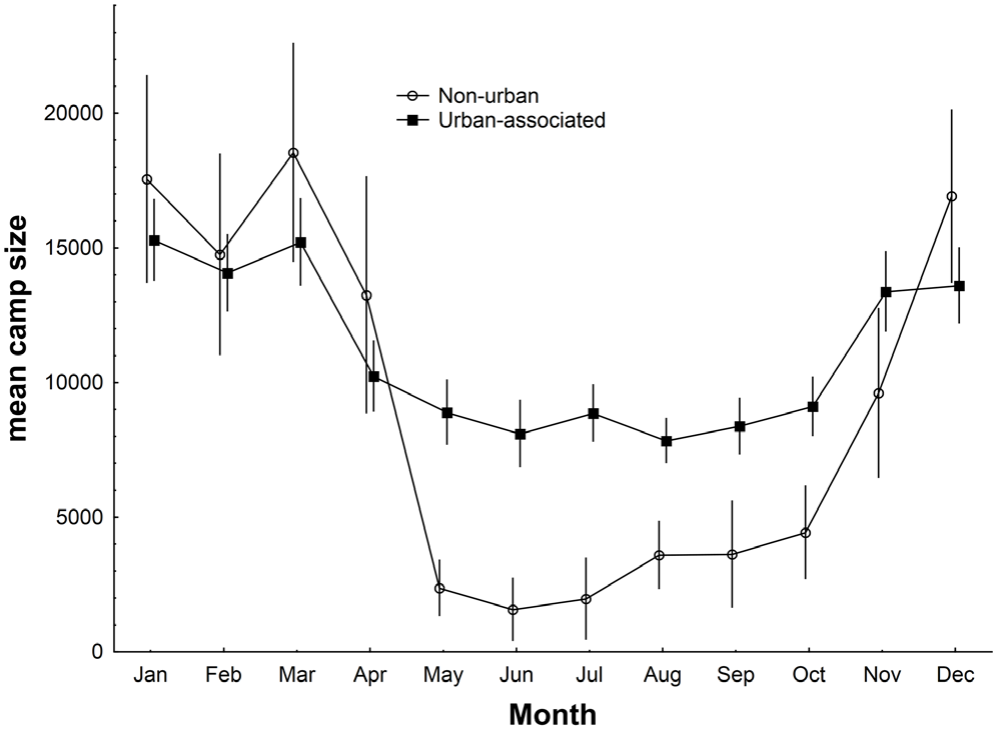
Changes through the year in the mean monthly camp size for urban-associated and non-urban camps. Calculated from 2004–2012 data.

There was a distinct seasonal pattern in the proportion of the population that was encountered in urban-associated camps with this proportion consistently reaching high levels in May and June before declining slowly there after (Figure 3). The proportion of the population found in urban-associated camps was greatest during the period of the year when the population count was at its lowest, May–November (Figure 4), and was significantly greater than during the rest of the year (Mann-Whitney U test, Z = −4.95, p<0.0001). During this May–November period all camp types were less consistently occupied (Mann-Whitney U test, Non-urban: Z = −3.56, p<0.05; Urban-associated: Z = −3.58, p<0.05), and had smaller mean camp sizes (Mann-Whitney U test, Non-urban: Z = 6.06, p<0.05; Urban-associated: Z = 4.64, p<0.05), though the decrease in non-urban camp size is far more pronounced than that of the urban camps (Figure 5). Urban camps were occupied in 32% of month’s surveyed (n = 16 camps), peri-urban camps 22% (n = 22) and non-urban camps 10% (n = 20).

**Figure 6:**
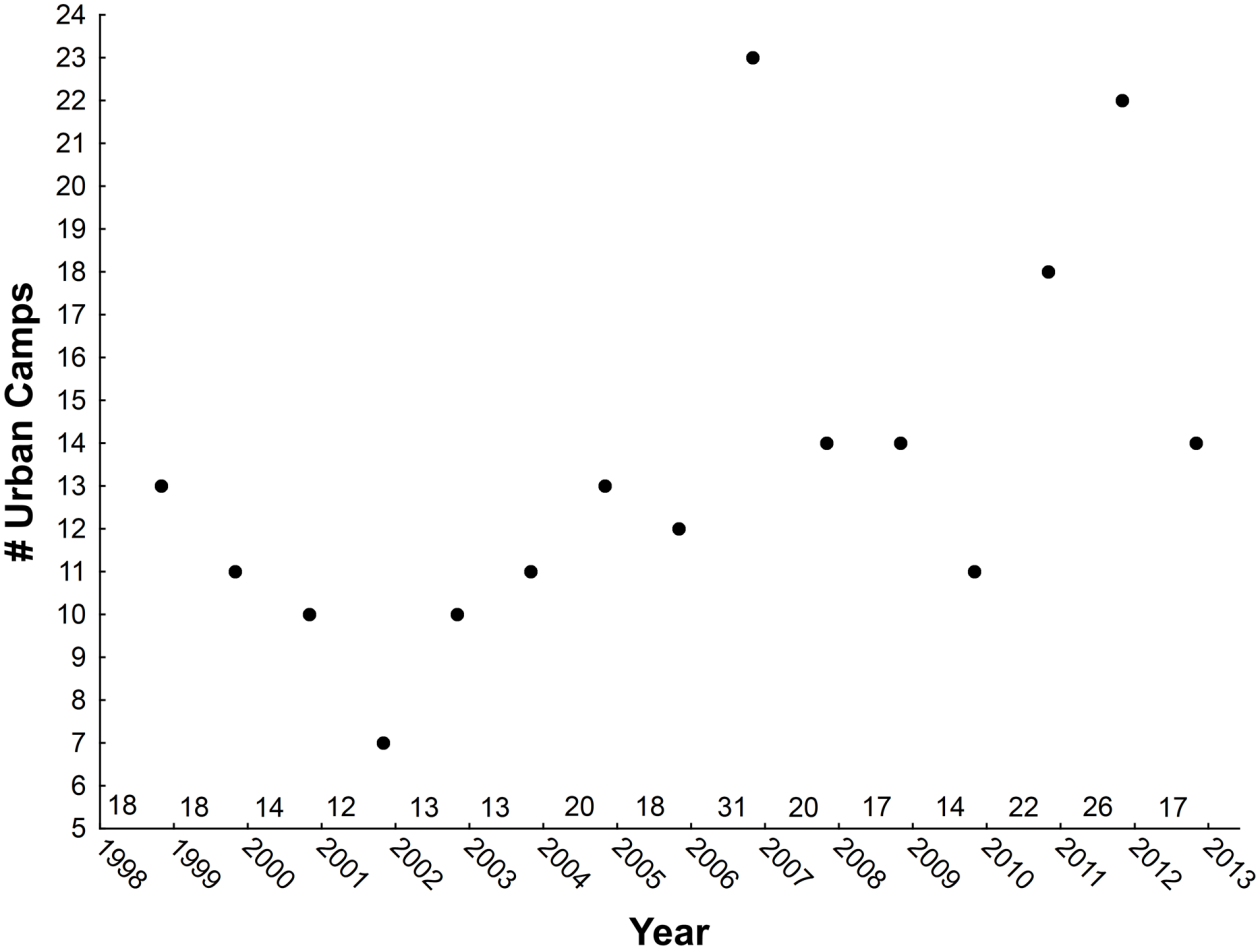
The number of urban associated camps recorded in surveys conducted in November of each year of the study. The number of occupied camps recorded in each year is indicated on the x axis.

There was an increase in the proportion of the population recorded using urban and peri-urban camps over the study period (Figure 2). This was the case irrespective of whether we considered the proportions recorded during November surveys only, the month that was surveyed in all years and in which the greatest proportion of the population are encountered in camps, (r_p_ = 0.69, p<0.005, n = 15), those for March and November surveys only, i.e. the months surveyed in all years except 2000–2003 (r_p_ = 0.54, p<0.01, n = 26), or for all months for which we have data (r_p_ = 0.28, p<0.005, n = 111). This trend appears to be due to a decrease in the number of non-urban camps used in any month (November only – r = −0.81, p<0.001, n = 15; March and November – r = −0.78, p<0.001, n = 26; all months – r = −0.57, <0.001, n = 111) and an increase in the number of urban camps used per month over time (November only - r = 0.7, p<0.002, n = 15, [Figure 6], November and March - r = 0.75, p<0.001, n = 26, all months – r = 0.56, p<0.001, n = 111).

**Figure 7:**
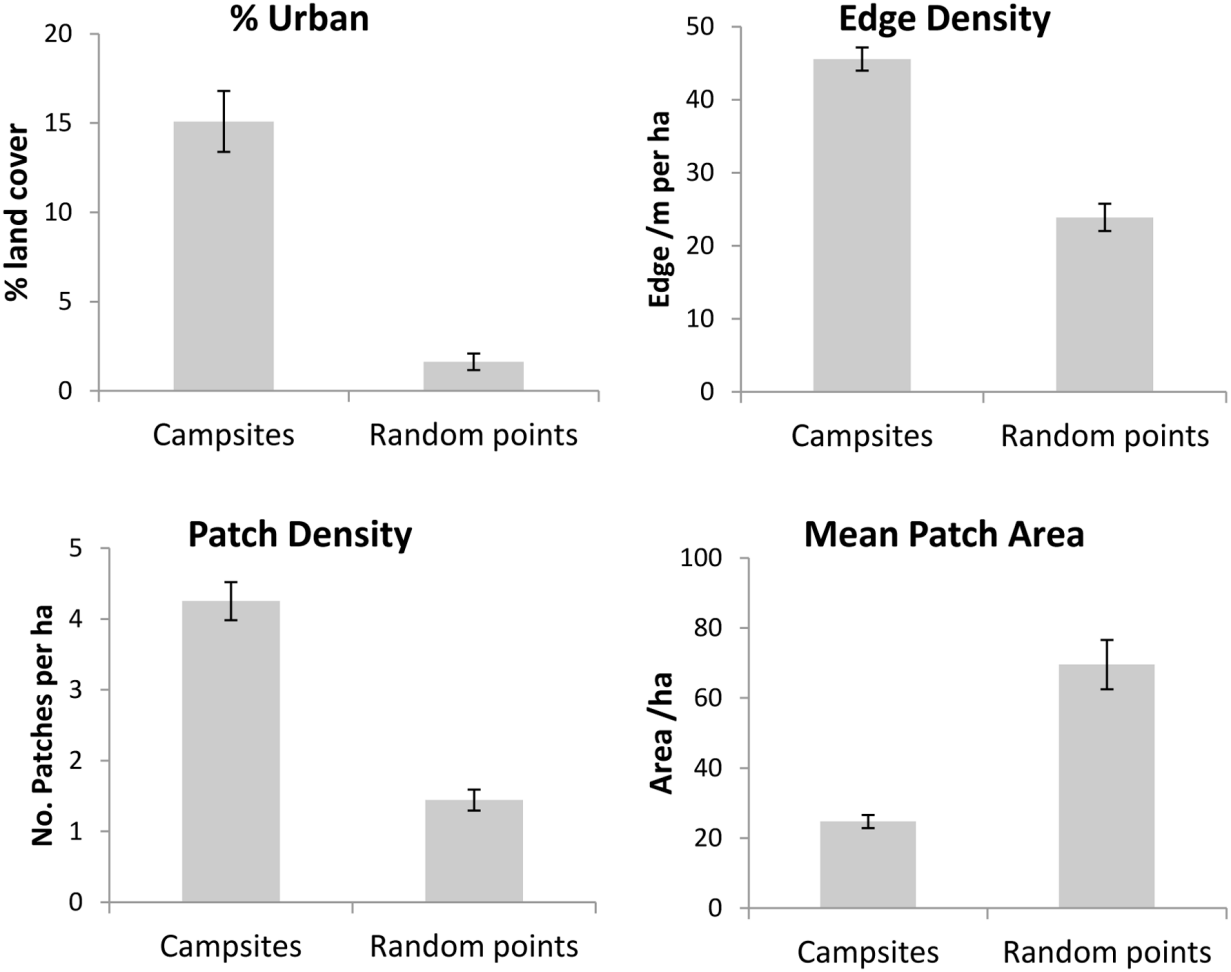
Landscape metrics (mean, ±SE) for buffer zones with a 3.3 km diameter surrounding all recorded campsites and random points in the landscape showing: percentage urban land cover, edge density, patch density and mean patch area.

A comparison of the landscape context of random points and the camps indicated that camps are significantly closer to urban areas than would be expected by chance (Wilcoxon signed rank test, Z = 33.26, p<0.0001). This effect did not arise during the study due to changes in where flying-foxes roosted. Individual camps did not move closer to urban areas over the period of the study. Nor did changes over time in the sub-set of camps occupied result in camps on average becoming closer to urban areas. Nor was the association of camps with urban areas due to urban expansion during the study. Urban land cover increased over the period of 1999 to 2009 from 1.3% to 1.4%, however this was not a statistically significant effect at the scale of the region (Table S2 in File S1). While there was a greater percentage of urban land cover around camps than around randomly chosen points in the landscape (Mann-Whitney U test, Z = 5.41, p<0.0001; Figure 7), comparison of land cover in 1999 and 2009 indicates no change in the extent of urban land use in the areas surrounding recorded camp locations (Mann-Whitney U test, Z = 0.31, p = 0.76). Similarly, areas surrounding occupied campsites did not become more fragmented over time than the randomly chosen locations, with no significant changes in MPA, PD or ED over time (p>0.05 in all cases). Despite this, camps do occur in locations that are more fragmented than random points in the landscape, with a smaller MPA, a significantly greater ED and PD (Mann-Whitney U tests, Z = 4.11, 3.54 and 4.55, respectively, p<0.001 for all; Figure 7).

## Discussion

We found that spectacled flying-foxes commonly roost near humans, as is the case for other *Pteropus* species in Australia, e.g. *P. poliocephalus, P. alecto, P. scapulatus* [20], [21], [23], and elsewhere, e.g. *P. giganteus* [45], and *P. dasymallus* [46]. Over the period of our study the majority of the spectacled flying-foxes found in camps were found in urban-associated camps, these camps were more consistently occupied than non-urban camps, and the proportion of the counted population encountered in urban camps increased. Furthermore, while the number of non-urban camps declined through the period of the study, the number of urban camps increased.

Of the hypotheses proposed for the association between flying-foxes and urban areas, our data only allow direct assessment of hypotheses related to the effect of changes in landscape structure. Given that we found i) no significant changes in land cover at the scale of the landscape as a whole or, ii) in the immediate vicinity of camps, and, iii) no change in the proximity of individual camps to urban areas over the duration of the study, our findings for spectacled flying-foxes do not support the hypotheses that urbanisation is occurring due to urban expansion towards camps, habitat loss, roost site loss or habitat fragmentation [20], [47], [48], [49]. Whether this also holds for other *Pteropus* species remains to be seen.

Our data does allow us to indirectly consider some alternative hypotheses for flying-fox urbanisation. Peaks in the urban-associated percentage of the population in 1998, 2000, 2003, 2006–7 and 2011–12 and are apparent in the November, November and March and all months’ samples. The peaks suggest that there are periodic shifts in the distribution of the population towards urban areas. Although the drivers of these temporary shifts are unknown, explanations could include the attraction of fruiting or flowering events near urban areas [24], or disturbance events such as droughts [28], fires [29], cyclones [22], [30], [31], and human disturbance or culling at non-urban roosts or orchards [24]. While there were two major cyclonic disturbances during the study period, in 2006 and 2010 and preceding the peaks in 2007 and 2011 respectively, no disturbance events can be associated with the other peak years. Significantly, these cyclones also precede peaks in the number of occupied camps (Table S1 in File S1). Furthermore, changes in permitting for mitigation of flying-fox damage in orchards mean that there has been a decrease in disturbance and culling at non-urban associated camps over the period of the study [37]. Rather than being driven by increasing or episodic disturbance, the long-term trend of increasing urbanisation since 1998 suggests a longer-term population shift towards urban areas is occurring.

Another explanation for these results could be bias in the sampling of urban and non-urban camps; urban camps are easier to locate, access and survey and therefore potentially more likely to be monitored. Such bias is unlikely to explain our results for the following reasons. First, camps are never dropped from our monitoring, even when they haven’t been occupied for long periods, so there is no shift of monitoring to an urban focus that might result from easier sampling. Second because non-urban camps are more likely to be overlooked than urban camps, we would expect an initial bias towards urban camps that would decrease as the population became better known through broad-scale searches, reports from the public [22] and telemetry studies [36], [50]. Instead we have seen the shift towards urban camps increase. Consequently, we feel confident that our results are not due to a sampling bias favouring urban camps.

Our data shows a seasonal pattern of change in the size of the urban-associated proportion of the population encountered in camps, with a greater proportion found in these camps from May–Nov than Dec-Apr (Fig. 4). This period is also when the population count is at its lowest, and camps of all types are less reliably occupied and have a smaller mean size. It is possible that without strong social reasons for aggregating (at this time mating has finished and females are pregnant), the animals disperse, possibly in association with reduced or more widely dispersed foraging resources. The decrease in non-urban camp size at this time of the year was far more pronounced than that of urban-associated camps, suggesting urban-associated camps may be core population centres (Fig. 5).

The urbanisation of spectacled flying-foxes documented here has significant implications for how the management of this, and other flying-fox species, is approached. Our results support the suggestion that flying-foxes are becoming increasingly urbanised and suggest that the conflict their presence in urban areas engenders is not going to go away. The lack of evidence for loss of habitat or roosting sites as a driver of this shift further suggests that spectacled flying-foxes are not being forced into urban areas, raising the possibility that their move is a behavioural response to the advantages offered by such locations. If this is the case then it is difficult to argue that moving problem urban camps on through the use of disturbance is likely to have any significant negative impacts on the population. Despite this, there is little evidence that past attempts to move urban camps have been successful or cost effective [23] and newspaper reports from Far North Queensland over the last century suggest that the common use of lethal methods was ineffective in deterring spectacled flying-foxes from urban areas (DAW, unpubl. data). We believe that this points to a need to explore new management options, particularly the identification of options that facilitate the co-existence of humans and flying-foxes. Management of the human side of the conflict is likely to prove more cost effective and successful. Identifying the actual drivers of urbanisation of flying-foxes will be significant for understanding and managing this process.

## Supporting Information

File S1 **Table S1.** Patterns of occupancy of camps of different types over the duration of the study. **Table S2.** Proportion of the landscape in each landcover category across the period of the study, 1999 and 2009. There are no significant differences between the proportions in each category in 1999 and 2009 (p = 0.05).
(DOCX)
