# Supplementary material for: Are Flying-Foxes Coming to Town? Urbanisation of the Spectacled Flying-Fox (Pteropus conspicillatus) in Australia
Source: PLoS One. 2014 Oct 8;9(10):e109810. doi: 10.1371/journal.pone.0109810 (PMC4190360; doi:10.1371/journal.pone.0109810)
Supplement: File S1 — Table S1. Patterns of occupancy of camps of different types over the duration of the study. Table S2. Proportion of the landscape in each landcover category across the period of the study, 1999 and 2009. There are no significant differences between the proportions in each category in 1999 and 2009 (p = 0.05). (DOCX) [file pone.0109810.s001.docx]

**Supporting Information**

**Table S1:** Patterns of occupancy of camps of different types over the duration of the study

| **Year** | **Non-urban** | **Peri-urban** | **Urban** | **Total** |
| --- | --- | --- | --- | --- |
| 1998 | 5 | 8 | 5 | 18 |
| 1999 | 7 | 7 | 4 | 18 |
| 2000 | 4 | 5 | 5 | 14 |
| 2001 | 5 | 5 | 2 | 12 |
| 2002 | 3 | 6 | 4 | 13 |
| 2003 | 2 | 6 | 5 | 13 |
| 2004 | 7 | 8 | 5 | 20 |
| 2005 | 6 | 7 | 5 | 18 |
| 2006 | 8 | 12 | 11 | 31 |
| 2007 | 6 | 7 | 7 | 20 |
| 2008 | 3 | 8 | 6 | 17 |
| 2009 | 3 | 5 | 6 | 14 |
| 2010 | 4 | 11 | 7 | 22 |
| 2011 | 4 | 10 | 12 | 26 |
| 2012 | 3 | 7 | 7 | 17 |

Table S2: Proportion of the landscape in each landcover category across the period of the study, 1999 and 2009. There are no significant differences between the proportions in each category in 1999 and 2009 (p=0.05)

| Landcover | %  1999 | %  2009 | χ^2^ |
| --- | --- | --- | --- |
| Cleared | 17.8 | 17.7 | 0.0003 |
| Other | 4.4 | 4.4 | 0.0000 |
| Rainforest | 24.7 | 24.6 | 0.0000 |
| Rural Residential | 0.8 | 0.9 | 0.0065 |
| Sclerophyll | 51.9 | 51.8 | 0.0000 |
| Urban | 0.5 | 0.6 | 0.0034 |
| Total | 100.0 | 100.0 | 0.0102 |
